# Bilirubin – new insights into an old molecule

**DOI:** 10.11613/BM.2025.020501

**Published:** 2025-04-15

**Authors:** Ivana Čepelak, Slavica Dodig, Ivan Pavić

**Affiliations:** 1 Department of Medical Biochemistry and Hematology, Faculty of Pharmacy and Biochemistry, University of Zagreb, Zagreb, Croatia; 2 Department of Pulmonology, Allergology, Immunology and Rheumatology, University Children's Hospital Zagreb, Zagreb, Croatia; 3 School of Medicine, University of Split, Split, Croatia

**Keywords:** antioxidants, bilirubin, cell signalling, immunomodulation

## Abstract

For decades, bilirubin was thought to be merely a degradation product of the hem, a potentially toxic compound and a molecule with no specific physiological function. Recently, it has been discovered that bilirubin has a strong antioxidant effect and possesses molecular signalling, hormonal and immunomodulatory properties. Numerous studies show that moderately elevated serum bilirubin concentrations correlate with a lower risk of developing pathological conditions mediated by oxidative stress and inflammation. Low concentrations within the current reference interval have been shown to be a potential risk factor for various pathological conditions. It is to be expected that knowledge about bilirubin will lead to the determination of bilirubin concentration gaining new importance in diagnostics, for example as a prognostic marker, but also as a therapeutic molecule.

## Graphical abstract

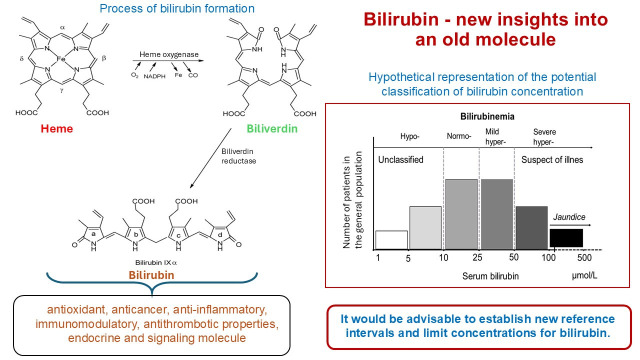

## Highlights

• Bilirubin is a potentially toxic molecule, but it also has physiological functions

• In some pathological conditions, a low bilirubin concentration may be a potential risk factor

• A slightly elevated serum bilirubin concentration may have health benefits

• It would be advisable to establish new reference intervals/limit concentrations for bilirubin

• Different limits could be investigated and/or established in relation to specific clinical conditions

## Introduction

For many years, the bilirubin molecule (C_33_H_36_N_4_O_6_) was considered only a waste product without a specific physiological function, a potentially toxic compound and a compound whose serum concentrations provide valuable information in the pathophysiology of liver diseases (*e.g.* viral hepatitis, autoimmune hepatitis, primary biliary cirrhosis, alcohol- or drug-induced toxicity, obstruction by gallstones, tumours, cysts) (*1*-*3*). In addition to this valuable information on the pathophysiology of liver diseases, Stocker and colleagues showed in 1987 that bilirubin has significant antioxidant properties (*4*). Later, other properties of unconjugated bilirubin (UCB) were discovered that depend on its concentration. These include anti-cancer, anti-inflammatory, immunomodulatory, anti-thrombotic and hormonal properties (*5*-*10*). Bilirubin has also been shown to inhibit lipid oxidation more effectively than vitamin E (*11*).

In the last three to four decades, it has also been found that bilirubin exhibits dual behaviour, that it has both potentially protective properties and toxic effects under certain pathological conditions. Since then, knowledge of the above functions of bilirubin has continuously stimulated research, including the evaluation of the clinical significance of serum bilirubin concentrations that are within or moderately above the valid reference interval (RI) of 3-20 μmol/L (*12*). Low serum bilirubin concentrations within the RI (< 10 µmol/L or < 7 µmol/L) are considered a potential risk for some diseases based on research findings (*10*). Moderately elevated concentrations are considered a predictor of and protective against diseases generally associated with increased oxidative stress, inflammation, metabolic disorders and an excessive immune response. These include, for example, cardiovascular diseases, autoimmune diseases, some cancers, arterial hypertension, diabetes, metabolic syndrome, obesity, *etc*. Most of these diseases are associated with the aging process and it has also been described that UCB can serve as a predictive biomarker for biological aging (*13*).

This review focuses on bilirubin as an antioxidant, immunomodulatory and signalling/hormonal molecule. It also addresses the potential clinical significance of low and moderately elevated serum bilirubin concentrations and bilirubin as a potential therapeutic molecule.

## Material and methods

A summary of the literature search data is presented in Table 1.

**Table 1:** Short overview of the literature search

**Item**	**Specification**
Timeframe of literature search	1938 - December 2024
Databases and other sources searched	Pubmed, Mediscape, Research Gate, Medline, National Library of Medicine and Academia.edu
Search terms used	bilirubin, hypobilirubinemia, antioxidant, hyperbilirubinemia, oxidative stress, signal molecule, Gilbert syndrome
Inclusion criteria of reference	language was restricted to English; articles involving bilirubin and its metabolic pathway; newly discovered properties of bilirubin; low and elevated serum bilirubin concentrations
Exclusion criteria of reference	articles involving severe hyperbilirubinemia
Selection process	all authors participated in the literature selection
Total number of articles 1938	160 1
Period 1987-2007	19
Period 2008-2020	56
Period 2021-2024	22
Number of included/excluded articles	98/62
The table contains an organised overview of all phases of the literature search. The time frame included published articles from 1938 to December 2024 in the selected databases. Of the 160 articles selected, a total of 98 were included and 62 excluded. The number of included articles by time period is also indicated.	

## Metabolic pathway of bilirubin

**Figure 1:**
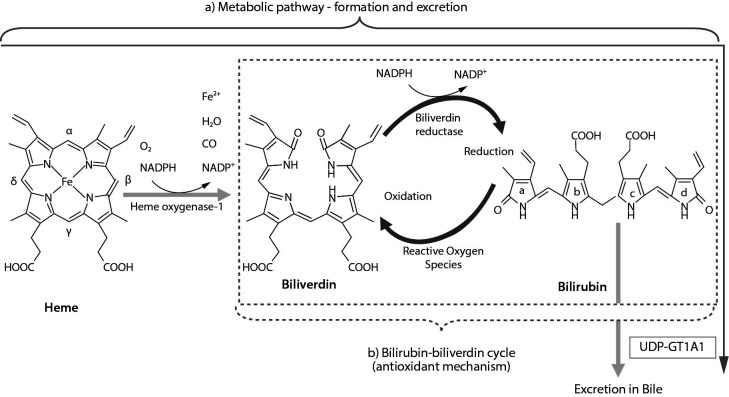
Metabolic pathway of bilirubin: a) formation and excretion process, solid line; b) bilirubin-biliverdin cycle (antioxidant mechanism), dashed line. NADP - nicotinamide adenine dinucleotide phosphate. NADPH - reduced nicotinamide adenine dinucleotide phosphate. UDP-GT1A1 - uridine diphosphate glucuronosyltransferases 1A1. Adapted from Reference 32.

Bilirubin is a molecule that occurs in all eukaryotes. It is one of the degradation products of the hem (Fe-protoporphyrin IX), a component of the hemoglobin molecule and other hemoproteins (*e.g.* myoglobin, cytochromes, catalase, peroxidase, tryptophan pyrolase, *etc.*) (*1*, *14*). Hem is released from hemoglobin after lysis of aged erythrocytes in the reticuloendothelial system together with globin. In free form and in large quantities, this tetrapyrrole compound is toxic, so that it is broken down very quickly thanks to the activity of microsomal hem oxygenase-1 (HO-1). This degradation process produces equimolar amounts of carbon monoxide (CO), water-soluble and relatively non-toxic biliverdin molecules and iron (Fe^2+^) as products, which leads to the induction of ferritin (*15*). Biliverdin is then reduced by the catalytic action of biliverdin reductase (BVR) to hydrophobic, water-insoluble and potentially toxic UCB, an open-chain tetrapyrrole (Figure 1). The structure of the molecule with six internal hydrogen bonds is responsible for the physicochemical properties and biological effects of UCB. All hydrophilic groups of bilirubin are involved in strong hydrogen bonds that lead to a closed, rigid conformation of the molecule with hydrophobic properties. For all three resulting molecules, iron, bilirubin and CO, desirable physiological and protective, but also undesirable toxic properties have been described, depending on the concentration (*16*). The unconjugated form of bilirubin enters the bloodstream, binds to albumin and can thus be transported to the hepatocytes. Under ideal conditions, there is no free UCB (unbound to albumin) in the serum/plasma. Entry into the cells occurs partly passively, but also with the help of *e.g.* the organic anion-transporting polypeptides 1B1 and 3, the multiple drug-resistant protein 1, *etc.* (*17*, *18*). Transport through the hepatocytes occurs by binding to glutathione S-transferase (or ligandin; limits the reflux of bilirubin into the bloodstream as the affinity for bilirubin is about five times higher than that for albumin) and to the Z-protein (fatty acid binding protein) under conditions of high bilirubin concentrations. This binding increases the uptake of UCB into the cell and minimises the efflux of internalised UCB back into the bloodstream (*14*).

Under normal conditions, almost all bilirubin is conjugated/esterified with glucuronic acid in the liver under the catalytic action of uridine diphosphate glucuronosyltransferases (UDP-GT) (*19*). There are several isoforms of UDP-GT, but the physiologically important isoform in bilirubin glucuronidation is the isoform UDP-GT1A1. Esterification breaks intramolecular hydrogen bonds, opens the molecule and makes conjugated bilirubin more hydrophilic so that it can be excreted into the bile. Conjugated bilirubin, together with bile acids and other components of bile, enters the intestine postprandially, where it is metabolised by intestinal bacteria (mainly of the genera *Clostridium* and *Bacteroides*) into metabolic products known as urobilinoids (urobilin, urobilinogen, stercobilinogen) (*20*). A small proportion of the urobilinogen formed is reabsorbed *via* the portal vein system and excreted in the urine, resulting in the colour of the urine. While the detection of urobilinogen in urine is a normal finding, the presence of bilirubin (conjugated form) in urine is always a pathological finding.

## The determination of bilirubin concentration

According to the Clinical and Laboratory Standards Institute, the reference method used in clinical practice is a spectrophotometric method based on the diazo reaction, in which azobilirubin is formed (absorbance at 550 nm) (*12*). This method is used as the gold standard, although the reaction can be disturbed, *e.g.* by immunoglobulins, hyperlipidemia, hemoglobin (hemolytic sample), vitamin C (*21*-*23*).

In most cases, the average total bilirubin concentration in serum/plasma in healthy people is around 10 μmol/L (normobilirubinemia). Literature data on values vary and reflect dependence on gender, age, ethnic group, circadian rhythm, time of sampling and other factors (*24*, *25*).

In brief, bilirubin, a by-product of hem breakdown, is mostly bound to albumin and conjugated with glucuronic acid in blood (< 5 µmol/L), whereas it is usually absent or present in negligible concentrations in free form (*12*). Usually, the concentration of total bilirubin (< 20 µmol/L) is determined.

## Bilirubin as an antioxidant

Although the antioxidant effect of bilirubin has been mentioned and demonstrated in numerous studies, the exact mechanism of action is still under investigation (*4*, *26*, *27*). The antioxidant property of bilirubin is related to its chemical structure - a linear tetrapyrrole, *i.e.* the exposure of hydrogen at the central C-10 position of the molecule (*4*). This hydrogen can bind to the outermost free electron pair of the oxygen radicals. In this way, it directly binds reactive oxygen species (ROS) (*28*).

Due to its lipophilic nature, UCB effectively interrupts the process of lipid peroxidation in cell membranes and prevents, for example, the oxidation of the low-density lipoprotein (LDL) molecule (*29*). It has an antioxidant effect in diseases associated with oxidative stress, *e.g.* diabetes, cancer, neurodegenerative and cardiovascular diseases and diseases of the reproductive system (*30*). The basic mechanism of bilirubin’s antioxidant action is thought to be related to the existing, evolutionarily conserved and energy-consuming (the formation of a UCB molecule costs the body a reduced nicotinamide adenine dinucleotide phosphate (NADPH) molecule) bilirubin-biliverdin cycle (Figure 1) and its excretion depends on conjugation by UDP-GT (*31*). When it acts as an antioxidant, bilirubin is oxidised to biliverdin, which is then reduced back to bilirubin by the catalytic action of BVR. The enzyme is present in considerable quantities in the cells. The substrate for BVR, which is formed by the action of ROS, is the isomer of biliverdin IXα. This is the phase of the reaction in which the energy resource is consumed in the form of NADPH (the electron donor in the reaction that produces bilirubin and nicotinamide adenine dinucleotide phosphate (NADP)). Lipophilic ROS act directly on bilirubin and lead to its oxidation to biliverdin.

It was also discovered that bilirubin activates the intranuclear translocation of the transcription factor nuclear factor erythroid-derived 2-like 2. This leads to an increased synthesis of HO-1 (as well as the formation of biliverdin and bilirubin) and antioxidants (*e.g.* superoxide dismutase, glutathione, *etc.*). This finding requires further research. It has been found that biliverdin also exhibits concentration-dependent antioxidant activity, albeit at a much lower intensity (*33*). The reconversion of biliverdin into bilirubin with the enzymatic property of enhancing the reaction can increase the antioxidant effect of bilirubin by a factor of 10,000 (*34*). In addition to the highly efficient suppression of the activity of *e.g.* singlet oxygen, superoxide anions and peroxyl radicals, bilirubin can directly and indirectly (by inhibiting the superoxide anion radical) inhibit peroxynitrite and thus prevent damage caused by this reactive compound (*35*). Although bilirubin is present in cells in nanomolar concentrations and acts by a different mechanism than the hydrophilic glutathione molecule, which is present in millimolar concentrations, the bilirubin-biliverdin cycle is thought to complement the glutathione system (*27*, *34*). The main feature and advantage of UCB as an antioxidant is its lipophilicity, which allows passive diffusion across cell membranes, including mitochondria (*36*).

In summary, a considerable number of experimental and clinical studies show that the reaction of conversion of biliverdin to bilirubin defines bilirubin as an antioxidant that protects cells from damage. This supports considerations of bilirubin as a prophylactic and therapeutic molecule in diseases with low bilirubin concentrations.

## Bilirubin as a signalling/hormonal molecule

The desirable effects of bilirubin, *e.g.* in obesity, diabetes, cardiovascular disease, at concentrations within or moderately above the RI (currently referred to in the literature as “high normal values”), were until recently mainly attributed to bilirubin’s action as a potent antioxidant (*34*). However, recent research has shown that bilirubin has the ability to bind directly/indirectly to cytoplasmic or nuclear receptors and activate other receptors depending on concentration (*37*, *38*). Such studies have defined bilirubin as a hormone and signalling molecule that initiates gene transcription and alters physiological responses (*10*, *39*). It has been described that, depending on the specific receptor, bilirubin can act as a ligand of biological targets *via* the receptor. Anti-inflammatory, anti-cancer, antihypertensive, anti-atherogenic, maintenance of energy homeostasis and other effects have been described (*40*-*42*).

Various modern experimental approaches (*e.g.* knockout animals, *in silico* modelling, PamGene technology, *etc.*) confirmed that UCB binds and activates the peroxisome proliferator-activated receptor-α (PPARα) at physiological concentrations (about 10-25 μmo/L) and thus triggers a gene response (*10*, *25*, *37*, *43*). This receptor, together with the other two, PPARγ and δ, belongs to the family of PPAR lipid sensors which, when activated by a ligand, regulate lipid and fatty acid homeostasis, *i.e.* energy storage and energy consumption (*44*). After entering the cell, presumably with the help of the fatty acid-binding protein transporter, bilirubin binds PPARα and induces corepressor proteins to convert into coactivators to activate genes for transcriptional control. The final metabolic effects of PPARα stimulation (not PPARγ and PPARδ), expressed in hepatocytes and adipocytes, among others, include increased expression of *e.g.* uncoupling protein 1, carnitine palmitoyltransferase and adrenocepto-beta 3, leading to desirable effects such as fat burning, weight loss and improved glucose homeostasis (*37*, *45*).

At high concentrations (> 200 μmol/L or > 150 μmol/L), which are usually measured in severe jaundice, UCB-bilirubin (but also conjugated bilirubin) has been found to react directly with *Mas*-related G localised on the cell membrane protein-coupled receptor member X4 (MRGPRX4) (*41*). The result is an enhancement of the downstream mechanisms of the itch receptor, *i.e.* intracellular calcium signalling in MRGPR-positive neurons that activate itch.

In addition to these two molecules, the literature also mentions other molecules that can be indirectly activated by bilirubin. These include, for example, the constitutive androstane receptor (recently identified as a therapeutic target for obesity and related metabolic disorders), PPARγ, the pregnane X receptor, the aryl hydrocarbon receptor, lipocalin-type prostaglandin D synthase and apolipoprotein D (*10*, *40*, *46*-*48*).

The protective effects of elevated bilirubin concentrations in combination with its properties as an endocrine and signalling molecule simultaneously indicate a potentially unfavourable role of low bilirubin concentrations in the pathophysiology of some human diseases. It is therefore necessary to continue research to clarify the role of bilirubin as a hormone/signalling molecule.

## Bilirubin as an immunomodulatory molecule

As a signalling molecule, bilirubin also has an inhibitory effect on the effectors of the immune system, which is expressed in bilirubin-mediated protection against autoimmune and inflammatory diseases (*49*). The first findings on the potential anti-inflammatory effect of bilirubin were made by Hench, who observed a temporary, significant remission of rheumatoid arthritis after the development of jaundice and a return of disease symptoms after the disappearance of jaundice (*50*). In later research, this effect was usually rightly attributed to the antioxidant property of the bilirubin molecule. Subsequent research has uncovered several other mechanisms of bilirubin’s anti-inflammatory effect.

Bilirubin has a complex immunosuppressive effect, which has been demonstrated in studies on animal models, but also to a lesser extent on human samples. Epidemiological studies have shown that elevated bilirubin concentrations are associated with a lower incidence of *e.g.* Crohn’s disease, multiple sclerosis and asthma (*51*-*53*). Moderate hyperbilirubinemia is associated with health benefits in overweight and obese individuals, diseases associated with low-grade chronic inflammation with increased oxidative stress, but also with altered endocrine signalling (*54*, *55*). An inverse relationship between C-reactive protein (CRP) and bilirubin concentration was also found in the serum of patients with diabetes and the inverse relationship between bilirubin and proinflammatory cytokines in serum (tumor necrosis factor-α, interleukin-6, visfatin, resistin - human model; monocyte chemoattractant protein-1 and leptin - animal model) and a positive relationship between bilirubin and adiponectin in humans with obesity (*56*-*58*). The bilirubin system has also been shown to be strongly induced in experimental autoimmune encephalomyelitis, an autoimmune disease mediated by T cells, *etc.* (*59*).

Almost a century after Hench’s publication, the mechanisms by which bilirubin in moderately elevated concentrations contributes to the alleviation of various inflammatory diseases were discovered. It has been experimentally demonstrated that bilirubin exerts its immunosuppressive effect by acting on granulocytes and lymphocytes, for example by inhibiting the cytotoxic activity of T lymphocytes (*60*). In an allergen-induced model of inflammatory colitis in mice, bilirubin has been shown to suppress inflammatory reactions by preventing the migration of leukocytes (especially eosinophil granulocytes) into the target tissue by interrupting cellular signalling that depends on the molecule vascular cell adhesion molecule-1 (VCAM-1) (*61*). A mechanism for modulation of leukocyte migration by bilirubin in response to VCAM-1 has also been proposed. Binding of the leukocyte VLA4 (a member of the integrin family α4β1) to VCAM-1 triggers activation of NADPH oxidase in response to calcium and Rac1. The result is the formation of ROS (superoxide, hydrogen peroxide), which stimulates the downstream activation of matrix metalloproteinases -2 and -9. Normally, the endothelial tight junctions are disrupted and the transmigration of leukocytes is facilitated. Fat-soluble bilirubin crosses the membrane and generates ROS *via* the intracellular bilirubin-biliverdin cycle, which are generated by the action of NADPH oxidase – endothelial retraction is inhibited and thus also leukocyte migration (*61*).

Li *et al.* have shown, for example, in their research *in vitro* (on cell inflammation models of mouse peritoneal macrophages and bone marrow macrophages), but also through tests *in vivo* (lipopolysaccharide-induced mouse sepsis), that bilirubin in physiological concentrations has a potential protective function (*62*). The protective mechanism includes inhibition of the nuclear factor kappa B signalling pathway and control of the activation of inflammatory mediators such as the NOD-like receptor protein 3 inflammasome. Wei *et al.* confirmed that bilirubin can attenuate cigarette smoke extract-induced mitochondrial dysfunction, cell necrosis and pyroptosis of macrophages (*63*). Bilirubin has also been shown to reduce the production of interleukin-2 in human lymphocytes, affect the expression of Fc receptors in macrophages, reduce the release of Damage Associated Molecular Patterns and interleukin-1β by injured pancreatic islet cells, which are an important trigger of the innate immune response after cell or organ transplantation, *etc.* (*7*, *64*, *65*). *In silico* research has shown that bilirubin can bind to the binding site of antigenic peptides of human leukocyte antigen molecules, preventing their binding to T cell receptors, which could lead to suppression of the autoimmune response (*66*).

Overall, the literature data from mainly experimental and clinical studies suggest that bilirubin is a molecule of immunological importance that shows a protective effect in inflammatory and autoimmune diseases.

## Clinical implications of low and slightly elevated (“high normal”) bilirubin concentrations

Until recently, lower and higher bilirubin concentrations within the above RI were not considered in relation to pathological conditions or specific symptoms. However, recent epidemiological and other studies have led to the following findings:

(a) Firstly - low bilirubin concentrations that fall within the lower quartile of the current RI for bilirubin (< 10 μmol/L or even < 7 μmol/L according to some authors), currently referred to as ‘’hypobilirubinemia’’, may be a potential risk factor for diseases associated with oxidative stress, inflammation and metabolic disorders (*10*). Such bilirubin concentrations have been shown to correlate with cardiovascular, brain-related and other complications (*67*, *68*). Higuchi *et al.* have shown, for example, that low bilirubin concentrations can be an indicator of an increased risk of white matter disorders of the brain in apparently healthy individuals (*69*). Another group of researchers has shown that low bilirubin concentrations are inversely correlated with common risk factors for cardiovascular disease such as cholesterol, body mass index (BMI) and type 2 diabetes (*70*). Lower bilirubin concentrations are also associated with systemic lupus erythematosus, Sjögren’s syndrome, rheumatoid arthritis, polymyositis and antiphospholipid syndrome, in which reduced bilirubin concentrations are closely associated with inflammatory markers (*71*-*75*). A negative relationship between total bilirubin and CRP, the erythrocyte sedimentation rate, has also been found in patients with inflammatory bowel diseases (ulcerative colitis and Crohn’s disease) and other pathologies (*76*). It was also found that patients with advanced renal dysfunction had significantly lower bilirubin concentrations than patients with normal renal function. This led the authors to conclude that “hypobilirubinemia” could be a potential risk factor for end-stage renal disease regardless of age, gender and estimated glomerular filtration rate (*77*). The cut-off concentration (or decision concentration) for “hypobilirubinemia” reported in the literature varies, so studies to establish a reliable concentration have not yet been completed (*78*).

b) The second finding comes from an extensive paper showing that bilirubin concentrations in the upper quartile of the RI and moderately elevated concentrations above the RI may be associated with the protective role of bilirubin against various diseases (*32*). The results of the study show that moderate unconjugated hyperbilirubinemia, which usually occurs in patients with Gilbert’s syndrome, has a protective effect. Gilbert’s syndrome, a hereditary, benign disorder, affects 3-7% of people worldwide and 5-10% in Europe. According to the literature, bilirubin concentrations in these patients vary between 20 and 70 μmol/L, depending on gender, age, ethnicity, smoking, diet and physical activity or, according to some authors, between 18 and 58 µmol/L (*38*, *39*). The condition is significantly associated with a lower incidence of *e.g.* diabetes, cardiovascular and neurodegenerative diseases, metabolic syndrome, which includes obesity, some cancers and lower mortality. Bilirubin-induced PPARα signalling is thought to be responsible for the significantly lower BMI, insulin and glucose concentrations in these patients. Thus, patients with Gilbert’s syndrome have significantly lower concentrations of many proatherogenic risk markers in lipid metabolism, including LDL, triacylglycerol and total cholesterol (*79*, *80*). They also have better antioxidant status and lower pro-oxidant and pro-inflammatory markers compared to individuals with normobilirubinemia (*79*, *81*, *82*). Slightly elevated UCB concentrations in RI have been shown to be beneficial for frequent wheezing in infants and childhood asthma. An *in silico* study found an inverse correlation of bilirubin with inflammatory mediators (*e.g.* leukotriene B4, arachidonic acid) and by-products of oxidative stress (*83*). By modulating signalling pathways critical to the development of these phenotypes, bilirubin has the potential to alleviate wheezing and reduce the risk of asthma in children.

According to the National Health and Nutrition Examination Survey, moderately elevated bilirubin concentrations appear to have a protective effect on the development of diabetes mellitus and stroke, which was also been confirmed by some other researchers (*84*-*86*). When the role of bilirubin was investigated in a mouse model with moderately elevated bilirubin concentrations, a protective effect against angiotensin II-dependent hypertension, a reduction in glomerular filtration and renal blood flow was found (*38*, *87*). The antioxidant activity of bilirubin was initially linked to its antihypertensive effect, but a more likely mechanism appears to be related to bilirubin’s properties as a signalling molecule, particularly as a selective PPAR modulator (*88*). Bilirubin has also been shown to be protective in various models of acute kidney injury (*89*). In the prospective Framingham Offspring Study, moderately elevated bilirubin concentrations were found to be associated with a lower risk of cardiovascular disease, coronary heart disease and myocardial infarction (*90*). According to the National Health and Nutrition Examination Survey conducted from 1999 to 2004 (a survey of 7075 patients), elevated bilirubin concentrations were associated with a lower prevalence of peripheral arterial disease (*91*). Patients with ovarian cancer who had higher bilirubin concentrations preoperatively were found to have higher overall survival and survival without disease progression than patients who had lower bilirubin concentrations before surgery, indicating a possible prognostic significance (*92*).

In summary, the results of large population-based, epidemiological and a larger number of studies in smaller subject groups show an association between the protective effect of a moderately elevated serum bilirubin concentration against cardiovascular and various metabolic diseases. Possible mechanisms of protective effects considered in various diseases include reduced formation of ROS (in the heart), effects on adhesion molecules (in the vessel wall), increased availability of nitric oxide (in the kidney), effects by which bilirubin acts as a signalling molecule (in the liver, in adipose tissue), *etc.* (*37*). Which of its properties (antioxidant, hormone, immunomodulatory) gives bilirubin a positive (preventive and protective) effect in the pathologies mentioned is currently being intensively investigated. In any case, the establishment of moderate hyperbilirubinemia is increasingly seen as an attractive strategy for the prevention of various diseases, especially those caused by oxidative stress or inflammation (*93*, *94*).

**Figure 2:**
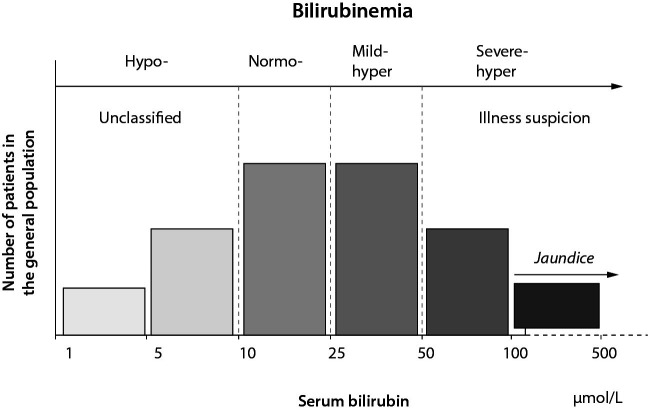
Hypothetical representation of serum bilirubin concentrations in general population and their potential classification. Adapted from Reference 37.

In line with the above research findings on low, “high-normal” and moderately elevated bilirubin concentrations, the research group of Creeden has constructed a theoretically possible representation of bilirubin concentrations in the general population that includes: a) values of 1-5 μmol/L, and b) concentrations between 5 and 10 μmol/L, which, although in the valid RI, are collectively classified as “hypobilirubinemia” for the time being; c) between 10 and 25 μmol/L or “high normal”, concentrations currently referred to as normobilirubinemia; d) 25 and 50 μmol/L represent the range of moderate hyperbilirubinemia, possibly related to health status; e) concentrations between 50-100 μmol/L represent true hyperbilirubinemia, and concentrations between f) 100 and 500 μmol/L represent severe hyperbilirubinemia (Figure 2) (*37*). The following studies should confirm or partially modify these ranges/concentrations, taking into account all possible factors influencing bilirubin concentration.

## Bilirubin as a therapeutic molecule?

In view of the numerous papers showing the inverse relationship between low concentrations and various diseases, and although a reliable threshold concentration has not yet been defined, numerous therapeutic strategies to increase bilirubin to moderate concentrations and strategies to promote stronger antioxidant, anti-inflammatory or immunomodulatory effects have already been considered in the literature. This is justified by the findings of Vitek *et al.* showing that even concentrations of a few μmol (about 1.7 μmol) increase in serum bilirubin reduce the risk of numerous pathological conditions (*39*).

Knowledge of the multiple functions of bilirubin, particularly its hormonal or signalling effects, has stimulated experimental research in this direction (*95*). The results of this research indicate the therapeutic potential of bilirubin (as a pretreatment or treatment) in the context of organ and tissue transplantation, diabetes and metabolic syndrome, acute kidney diseases, and topical application is being tested as an aid in wound healing. Research also includes the evaluation of potential therapeutic doses of bilirubin.

Although the insolubility of bilirubin and its susceptibility to oxidation complicate its use as a therapeutic molecule, research is continuing. Based on experimental research, non-pharmacological and pharmacological strategies are being considered. Non-pharmacological strategies include influencing bilirubin concentrations through lifestyle changes, *e.g.* maintaining an appropriate body weight, aerobic activity, dietary supplementation with plant polyphenols, flavonoids, *etc.* Pharmacological strategies include the use of bilirubin as a pharmacological molecule. There are various approaches, such as the induction of HO-1 by providing substrates such as hemoglobin or myoglobin; the administration of biliverdin as a precursor of bilirubin; UDP-GT inhibitors that suppress the natural conjugation of bilirubin in the liver; natural bilirubin of animal origin and synthetic analogues of bilirubin (*e.g.* cyanobacterial phycobilins with bilirubin-like properties) and, more recently, bilirubin encapsulated in nanoparticles. In 2016, Lee’s research group published two papers for the first time in which they used PEGylated bilirubin nanoparticles to treat cancer and chronic inflammation (*93*). All approaches are still in the preclinical phase of testing in cell, culture and animal models and will be continued – for a more detailed insight, see the review by Adin (*96*).

There are only a small number of tests in human samples with an acceptable safety profile and in a short time frame (*97*). The classes of drugs already used in clinical practice are also being investigated (*e.g.* non-steroidal anti-inflammatory drugs, hypolipidemic drugs, anti-aggregation drugs, H2 antihistamines and some antihypertensive drugs, as well as natural substances used as nutraceuticals (*39*). For example, it has been shown that patients infected with the human immunodeficiency virus and treated with atazanavir, which competitively inhibits the process of bilirubin conjugation, have a slight increase in bilirubin and a lower cardiovascular risk (*98*). Currently, there are no approved specific therapies to increase low bilirubin concentrations, but the results of numerous studies to date point in this direction.

In short, a sufficient number of population studies and clinical trials point to the dual role of bilirubin. One possible role of a low bilirubin concentration is that of a risk factor for the development of certain diseases. Therefore, both prophylactic and therapeutic strategies of bilirubin administration are being considered. The second aspect is the potentially protective role of bilirubin in moderately elevated, physiologically non-toxic concentrations. Based on these data, a reclassification of the RI for bilirubin and the concentration defining the terms “hypobilirubinemia“ and "hyperbilirubinemia" is considered and recommended. In this context, appropriate diagnostic specificity and sensitivity for certain conditions should be considered.

## Final considerations

Research in recent decades has expanded the understanding of bilirubin, particularly as a non-functional, potentially toxic waste product of metabolism and, in elevated concentrations, as an indicator of liver disease. There is ample evidence that bilirubin is a molecule with multiple biological functions, particularly antioxidant, signalling/hormonal and immunomodulatory effects.

In parallel with the discovery of new functions of bilirubin, the results of an increasing number of population studies and clinical trials point to the dual role of bilirubin in the context of various diseases - the role of a potentially protective but also a risk factor. This type of study is motivated by strong evidence that the moderate hyperbilirubinemia that occurs in individuals with Gilbert’s syndrome correlates with increased antioxidant status and decreased levels of pro-oxidant and pro-inflammatory markers. On the other hand, low bilirubin concentrations have been shown to correlate with a potentially higher risk of *e.g.* cardiovascular and metabolic diseases. According to the results of these researchers, *i.e.* the potential relevance for human health, different therapeutic approaches with natural compounds and synthetic drugs are proposed. However, it is necessary to verify their clinical benefits and toxicity risks.

Despite numerous papers demonstrating these associations, the mechanisms by which bilirubin provides protection or plays a role in disease development are only partially elucidated and further research with this aim is needed. In order to assess the clinical significance of the above research findings, new RIs for normobilirubinemia need to be established that take into account all important influencing factors. In addition, it is necessary to consider the cut-off concentrations of bilirubin for “hypobilirubinemia”, below which there is a risk of developing pathological conditions. In addition, the concentration above which the toxic effect of bilirubin occurs must be defined and the range of protective bilirubin concentrations must be established.

How this field, which some refer to as “bilirubinomics”, will evolve depends on ongoing research and future well-planned experimental and clinical research, which is definitely needed. Collaboration between chemists, biologists, physiologists, biochemists, physicians, biostatisticians, bioinformaticians and other professional profiles is necessary for the correct interpretation and definition of consistent conclusions and recommendations.

## Data Availability

No data was generated during this study, so data sharing statement is not applicable to this article.
